# The Asthma Phenotype in the Obese: Distinct or Otherwise?

**DOI:** 10.1155/2013/602908

**Published:** 2013-06-25

**Authors:** Sherry Farzan

**Affiliations:** North Shore-Long Island Jewish Health System, Division of Allergy and Immunology, 865 Northern Bouelvard Suite 101, Great Neck, NY 11021, USA

## Abstract

Asthma is a heterogenous disorder that can be classified into several different phenotypes. Recent cluster analyses have identified an “obese-asthma” phenotype which is characterized by late onset, female predominance and lack of atopy. In addition, obesity among early-onset asthmatics clearly exists and heightens the clinical presentation. Observational studies have demonstrated that asthma among the obese has a clinical presentation that is more severe, harder to control, and is not as responsive to standard controller therapies. While weight loss studies have demonstrated improvement in asthma outcomes, further studies need to be performed. The current knowledge of the existence of two obesity-asthma phenotypes (early- versus late-onset asthma) should encourage investigators to study these entities separately since just as they have distinct presentations, their course, response to therapies, and weight loss strategies may be different as well.

## 1. Introduction

Although the clinical definition of asthma, a chronic inflammatory disorder of the airways characterized by bronchial hyperresponsiveness and airway obstruction resulting in respiratory symptoms, is uniform in the over 300 million individuals affected worldwide [1], clinicians diagnosing, treating and managing asthma can unequivocally agree that it is a heterogeneous disease. The clinical spectrum of disease, inflammatory milieu, demographic characteristics, and comorbidities define the asthma phenotype. Phenotype, defined as the set of observable characteristics of an organism that are produced by the interactions of the genotype and the environment, in asthma can be defined based upon a number of factors, including age of onset, atopy, inflammatory infiltrates, severity of disease, response to standard medications as well as a number of other factors. Delving deeper into the pathophysiology of the disease and the resulting endotype of asthma may help to tailor clinician's management of these diverse presentations of a single disease. The clinician's experience of such a diversity of asthma presentations has been supported by a plethora of observational and clinical studies as well as a number of recent cluster analyses which use statistical methods to categorize asthmatics based upon clinical and demographic factors. One phenotype which has been suggested in clinical studies and consistently identified in cluster analyses is that of an “asthma-obesity” phenotype. With the rising epidemics of both asthma and obesity in the United States, the “asthma-obesity” phenotype is attracting more attention and will require greater focus in order to appropriately and effectively identify and manage these individuals.

Large-scale epidemiological data demonstrate that obesity increases the prevalence of asthma [2] as well as the risk of incident asthma [3–6]. A large proportion of asthmatics are either overweight or obese. Data from the 2000–2004 NHANES, demonstrate that approximately 30% of adult asthmatics are overweight and an additional 30% are obese [7], with similar prevalence among pediatric asthmatics [8]. More recent data from clinical trials suggest that 50% of asthmatics are obese (personal communication, Anne Dixon). Furthermore, a number of studies have shown that obesity affects asthma severity [9] control [9, 10], and medication response [11–14]. This review will discuss the clinical dimensions of the “asthma-obesity” phenotype and will also discuss whether the “asthma-obesity” phenotype itself is heterogeneous. 

## 2. Cluster Analyses

Cluster analysis uses multivariate mathematical algorithms in order to quantify similarities between individuals within a population based upon multiple specific variables and then groups individuals into clusters based upon their similarities [15]. Haldar et al. were the first to apply this method to three populations of asthmatics in order to identify clinical asthma phenotypes [16]. Subjects were analyzed from three different populations: predominantly mild to moderate asthmatics managed in primary care, refractory asthmatics managed in secondary care and a third group of refractory asthmatics in a prospective clinical trial. In all three populations, a group of obese, predominantly female individuals with asthma were identified. These individuals had a high level of symptom expression in the absence of eosinophilic airway inflammation. Compared to the other clusters identified in the primary care cohort (early-onset, atopic and benign asthma), they had a later-age-of asthma onset, a lower level of atopy and fraction of exhaled nitric oxide (FeNO), moderate level of airway hyperreactivity and FEV1 reversibility, and highest level of airway neutrophilia and symptom expression (as assessed by a modified Juniper Asthma Control Score, mJACS). Although not reaching statistical significance, obese-asthmatics were on the highest doses of inhaled corticosteroids (ICS). In the secondary care cohorts, age of onset, atopic status and FeNO were similarly distinguishable in this cluster, however airway neutrophilia was consistently high in all clusters identified and symptom expression was not as high as in early-onset atopics. In the refractory subjects, the obese-asthmatics, along with the early-atopics did require the highest doses of ICS, however this reached statistical significance only in the retrospective group [16]. 

A cluster analysis from the Severe Asthma Research Program similarly identified a group of obese, predominantly female, late-age-of-onset asthmatics. Similar to Haldar's analysis, these individuals were less likely to be atopic. Despite mildly decreased baseline pulmonary function, these subjects tended to require more intensive medical regimens, including a higher rate of daily systemic steroid use, greater health care utilization, and had a high level of symptom expression that seemed to be out of proportion to their degree of airway obstruction. Furthermore, in a post hoc analysis, serum IgE levels were the lowest and sputum eosinophils and neutrophils were the highest in this obese-asthmatic cluster [17]. 

## 3. Early-Onset versus Late-Onset Asthma among the Obese

Nevertheless, obesity exists in a growing population of children and adolescents who have developed “early-onset” asthma. Holguin et al. addressed the question of how obesity may affect the presentation of asthma dependent on age-of-asthma onset, as opposed to those identified in the cluster analyses by Haldar et al. and Moore et al. Among both early- and late-onset asthmatics, a larger proportion of obese/overweight individuals were taking controller medications compared to their lean counterparts. Early- and late-onset obese asthmatics also had more continuous respiratory symptoms and lower quality of life compared to their lean counterparts. In addition, with increasing BMI, reduced FEV1 and FVC and greater airway obstruction were present only in the early-onset group. Early-onset obese asthmatics had more asthma morbidity and greater health care utilization in all categories assessed (OCS tapers, ED visit, hospital admission, ICU admission, mechanical ventilation and pneumonia diagnosis) compared to lean counterparts. Late-onset obese asthmatics had greater ER and ICU visits only, compared to lean counterparts [18]. 

When comparing obese/overweight asthmatics based upon age of onset, the early-onset group was younger, had a greater proportion of males, and more airway obstruction, FEV1 reversal, airway hyperresponsiveness and higher serum IgE levels (latter two parameters tested in a subset). In terms of morbidity, compared to the late-onset group, the early-onset asthmatics had a trend towards more steroid tapers, and ICU admissions did reach statistical significance. Furthermore, they found that early-onset asthmatics gained weight at a higher rate after the diagnosis of asthma than that in late-onset asthmatics [18].

Despite the earlier cluster analyses which identified the late-onset, female predominant “asthma-obesity” phenotype, and the majority of studies indicating that asthma in the obese has a more severe presentation, there have been reports in the literature of heterogeneous effects of obesity on asthma control. Therefore, Sutherland et al. performed a cluster analysis of a prospective trial of persistent asthmatics and found four unique clusters of asthma patients. Given that the mean BMI of the study population was 29.9, the mean BMI of two clusters fell in the overweight range while that of two fell in the obese range. Early age of asthma onset characterized the more severe obese group with greater airway hyperresponsiveness, inflammation (as indicated by FeNO), symptom expression, and suboptimal asthma control obtained by ICS. Otherwise, both obese age-of-onset groups had similar lung function impairment, atopy, adipokines and markers of systemic inflammation [19]. Although not addressing age-of-asthma onset, Lang et al., concluded that obesity had a greater detrimental effect on the lung function of children and adolescents as opposed to adults [20].

## 4. Obesity and Asthma Severity and Control

Although the majority of studies assessing asthma severity and control indicate a greater burden of disease among the obese, not all studies are consistent. In a cohort of patients from the National Asthma Survey, obese asthmatics were more likely to have severe persistent asthma (based on GINA classification) and poorer indices of asthma control (more respiratory symptoms, healthcare utilization, and short-acting beta-agonist use) [9]. However, Peters et al. did not find greater asthma severity or health care utilization among both obese children and adults, although quality of life was decreased among adults [21]. In addition to the findings of the cluster analyses discussed above [16–19], quality of life, symptom scores, rates of exacerbations, pulmonary function parameters and medication use tend to be higher among obese adults and children in a number of studies [10, 22–25]. In a cohort of severe asthmatics, obese individuals were less likely to be atopic and had greater use of maintenance and rescue oral corticosteroids as well as SABA, although there was no difference in ICU admission, ER or urgent visits [26]. Furthermore, Haselkorn et al. followed a group of asthmatics over one year and found that those who gained five pounds or more reported worse asthma control than steady weight or weight loss groups [27]. Nevertheless, even among these studies, some parameters were not significantly different between obese and non-obese individuals. For example, there was no difference in hospital-based health care utilization between obese and nonobese asthmatics in a prospective cohort at Kaiser Permanente [10] or in airway function and symptom scores in a smaller prospective study [25]. 

## 5. Obesity and Airway Physiology in Asthma

Increased BMI is associated with a number of physiologic changes in the airways, independent of asthma. The most dramatic effects of BMI are reductions in functional residual capacity and expiratory reserve volume. There are also mild reductions in total lung capacity, vital capacity and residual volume, which are seen primarily when comparing normal weight to those with BMI of 30 or greater. Proportional decreases in both forced expiratory volume in one second and functional residual capacity, results in a normal or restrictive airflow pattern [28]. Obesity itself is not associated with airflow obstruction [29]. However, since obese individuals do breathe at lower lung volumes, the resulting airway narrowing may contribute to heightened airway hyperreactivity [30–32], with one study noting the association only in females [33]. This latter observation may be due to a subgroup effect among the late-onset non-atopic obese asthma phenotype described above, as Dixon et al. [34] demonstrated that obese asthmatics who had a low serum IgE (presumably late-onset of asthma) had improved airway hyperreactivity 12 months after bariatric surgery, but those with elevated serum IgE (presumably early onset of asthma) did not. A heterogenous obese asthmatic population may explain why another study has not found a significant effect of obesity airway hyperreactivity [35].

## 6. Obesity and Airway Inflammation in Asthma

A number of studies have demonstrated that the airway inflammation associated with obesity-associated asthma is not the typical eosinophilic inflammation seen in early-onset atopic asthma. On the contrary, a number of studies have shown that BMI and/or waist circumference is inversely associated with sputum eosinophilia [24], FeNO [36], a noninvasive marker of eosinophilic inflammation, or both [37]. Meanwhile, other studies, which may have looked at a heterogenous group of obese asthmatics, did not note any relationship between obesity and sputum eosinophils [38] or FeNO [39]. These observations support the findings of the cluster analyses described above which identified a late-onset noneosinophilic phenotype of asthma to be associated with obesity.

Neutrophil-predominant airway inflammation, which is associated with more severe phenotypes of asthma, may play a greater role in obesity-associated asthma. Several studies have shown that airway neutrophils were increased in obese women with asthma [40, 41] One of these groups demonstrated in a follow-up study, that weight loss (through dietary restriction, with or without exercise) was associated with a reduction in airway neutrophilia [42]. These findings are not consistent with the cluster analyses discussed above in which airway neutrophilia was not a distinguishing feature in the obese asthmatic group, and eosinophilia was also present [16, 17].

There is some data to suggest that the “obese-asthma” phenotype may be associated with alterations in airway lymphocytes. Th2 CD4+ T helper cells are instrumental in the pathophysiology of typical allergic asthma, by elaborating cytokines that promote eosinophil infiltration and mucous production. As described above, the late-onset “obese-asthma” phenotype seems to be characterized by a non-eosinophilic infiltrate. In fact, Dixon et al. reported that bariatric surgery in obese asthmatics actually enhanced the airway lymphocyte numbers and peripheral blood lymphocyte proinflammatory cytokine elaboration, even when controlling for the use of inhaled corticosteroids [34], suggesting that the inflammation involved in the “obese-asthma” phenotype is not lymphocyte driven and thus can not be expected to respond dramatically to the standard lympholytic therapies used for asthma. In a mouse model of asthma, those mice which were given a high-fat diet similarly developed reduced lung eosinophilia and IL-5 [43].

Another marker of airway inflammation, 8-isoprostanes, have been found to be elevated in obese asthmatics when assessed by exhalation [36], but not in the BAL supernatant [44]. Leukotrienes, a group of proinflammatory lipid molecules which mediate bronchoconstriction in asthma, have also been linked to increasing BMI. The ratio of urinary leukotriene E4 to creatinine has a significant positive association with BMI in asthmatic subjects [45]. Leptin, levels of which are elevated in obesity, have been demonstrated to act on airway macrophages in mouse models and enhance leukotriene production [46].

## 7. Obesity and Asthma Therapy

With multiple lines of evidence suggesting that the “asthma-obesity” phenotype presents with greater severity, worse control, and altered inflammatory mechanisms, the therapeutic approach may need to be rethought as well. One of the earliest studies to suggest that standard controller medications are not as effective among obese asthmatics was by Peters-Golden, in which he demonstrated that rising BMI was associated with decreased effectiveness of inhaled corticosteroids, as assessed by the number of asthma control days. In contrast, increasing BMI did not alter the number of ACD in the group treated with montelukast [11]. In two other post hoc analyses, comparing ICS or ICS/LABA versus montelukast among the overweight and obese, ICS and ICS/LABA were consistently more effective than montelukast in all BMI catergories [46, 47]. In the study by Sutherland et al., the effectiveness of ICS did not decrease in a statistically significant or patient-centered manner [46]. Boulet and Franssen demonstrated that using a long-acting beta-agonist in addition to ICS was superior to ICS alone in all BMI groups, but the effectiveness of both treatment regimens decreased with increasing BMI [12]. When assessing how BMI affects controller responses in terms of asthma outcomes, Sutherland observed that ICS use more effectively decreased FeNO in lean versus overweight/obese asthmatics and ICS/LABA improved pulmonary function parameters (FEV1, FEV1/FVC, PC20 FEV1) more in lean versus overweight/obese asthmatics [13]. 

The cause for differential effects of ICS based on BMI has been hypothesized to be either mechanical (poor airway penetration in obese state) or molecular. Mitogen-activated protein kinase phosphatase-1 (MKP-1) is a molecule that mediates the negative regulation of the pro-inflammatory MAPK pathways by glucocorticoids. Sutherland et al. demonstrated that MKP-1 expression was reduced in dexamethasone-induced peripheral blood mononuclear cell (PBMC) and bronchoalveolar lavage cell of overweight/obese compared to lean asthmatics [14], suggesting greater glucocorticoid insensitivity in the former group. The altered molecular fingerprint in the overweight/obese asthmatics is purported to be due to the inflammatory milieu propagated by adipose tissue [48].

## 8. Asthma and Weight Loss

With studies showing limited effectiveness of standard asthma therapies, a more tailored approach must be identified to manage obese asthmatics. There are multiple studies addressing the issue of medical or surgical weight loss in improving asthma endpoints among overweight and obese individuals. The first of these studies, by Stenius-Aarniala et al. randomized 38 obese subjects with asthma to a supervised weight reduction program including a very low-calorie diet or control. Compared to control, the intervention group had significant improvements in symptoms, lung function, and health status [49]. Since such strict dietary interventions are difficult to maintain, Johnson et al. assessed the effect of an 8-week alternate day calorie restriction diet on obese individuals with asthma and found that there were significant improvements in asthma control, symptoms, quality of life, peak flow, airway reversibility and markers of oxidative stress and inflammation compared to baseline [50]. In another study, which followed a cohort of obese women with asthma who underwent a six-month dietary weight loss program, there were improvements in pulmonary function and disease-specific quality of life, but no changes in airway hyperreactivity compared to baseline. This study, similar to the one previously mentioned, did not have a control group, and included individuals without a physician diagnosis of asthma [51].

Studies looking at the effects of bariatric surgery on various comorbidities, including asthma [52–54], or specifically at asthma [55–58] have all reported improvements in various asthma endpoints. In a nonblinded, nonrandomized study of obese asthmatic females, 12 underwent laparoscopic adjustable gastric banding and were found to have significant improvements in ACT scores, shortness of breath, rescue medication use and PFT parameters, whereas none of the subjects in the control group did. While there were no differences in exhaled nitric oxide before and after surgery in this study [55] another prospective study did find that there were significant reductions in exhaled nitric oxide in obese asthmatics one year after bariatric surgery [56]. The majority of asthmatics in this study were atopic. In a large retrospective study, 2,562 patients undergoing bariatric surgery reported using asthma medication at baseline. At one year after surgery, 257 of these patients participated in a follow-up and reported a decrease in controller medication use (oral/inhaled corticosteroids, inhaled bronchodilators), with nearly 40% needing no medications for asthma at one year. Interestingly, those individuals who underwent laparoscopic adjustable gastric banding, which was associated with less dramatic weight loss than other bariatric surgery techniques, had less improvement in asthma endpoints [57]. In a prospective study of 23 (12 undergoing bariatric surgery) obese asthmatics, airway hyperreactivity, pulmonary function and asthma control were assessed at six and twelve months. While no significant changes were observed among the controls, those who underwent bariatric surgery had significant improvements in airway hyperreactivity, pulmonary function and asthma control. While this study did not note a difference in changes of airway hyperreactivity based on atopy [58], a similar study by Dixon et al. found that only non-atopic (low IgE, presumably late-onset asthma) obese asthmatics had improved airway hyperresponsiveness after bariatric surgery [34]. Differences in their findings may be due to sample size. In Dixon's study, there was also an unexpected increase in bronchoalveolar lavage lymphocytes and peripheral CD4+ T cell cytokine production after bariatric surgery [34]. 

Three systematic reviews of medical and surgical weight loss studies have been published addressing this issue. In two of the reviews, although the authors note that despite the heterogeneity in the populations, interventions and outcomes studied and the fact that an asthma endpoint was not the primary outcome in most of the studies, there was improvement in at least one asthma outcome (asthma symptoms, severity, control, medication use, rates of exacerbation, PFT parameters, and airway hyperreactivity) in all of the studies [59, 60]. A Cochrane review, however, which analyzed the only four randomized controlled trials assessing the impact of weight loss and asthma, found improvement in asthma control in only one study and came to the conclusion that due to the low quality of evidence (due to bias and imprecision), the benefit of a weight loss intervention to improve asthma among overweight and obese individuals is uncertain at this time [61].

## 9. Obesity and Pediatric Asthma

While children with asthma who are obese, due to the timing of asthma incidence, would theoretically fall into the early-onset asthma phenotype for the most part, there have been some studies examining the relationship more closely and there is a thorough review of the topic by Jensen et al. [62]. An analysis of the 1999–2006 NHANES data demonstrated that obesity was more significantly associated with nonatopic asthma, as opposed to atopic asthma in obese children [63]. Similar to adults, asthma control deteriorates and asthma severity escalates with increasing BMI in most studies [8, 64, 65]. In terms of airway physiology in the obese asthmatic child, the data is conflicting. While some studies report no difference in lung function parameters between obese and normal weight asthmatic children [21], other studies do demonstrate significant differences [66–68], especially with respect to a reduced FEV1/FVC ratio for children with elevated BMI. Studies addressing airway inflammation in obese asthmatic children are lacking, but when using FeNO as a marker of eosinophilic inflammation, it is not associated with elevated BMI [8]. Similar to adults, the efficacy of inhaled corticosteroids is reduced in the obese asthmatic children [67]. 

A few medical weight loss studies have been performed in the pediatric population. In one study, in which pulmonary function tests were performed in 20 obese children with asthma before and after a weight reduction program, investigators found that improvements in FEV1 and FVC correlated with BMI and other anthropometric measurements [69]. In a larger study of 76 adolescents (26 asthmatics), a one-year interdisciplinary weight loss intervention resulted in improvements in lung function and adipokine profile in both obese asthmatics and nonasthmatics, with changes in adiponectin levels predicting improvements in lung function. Furthermore, among the asthmatic subjects, there was a reduction in asthma severity, asthma symptoms, and rescue medication use [70]. The same group reported that an interdisciplinary intervention reduced exercise-induced bronchospasm frequency and resulted in a more favorable adipokine profile among a smaller group of obese adolescents with exercise-induced asthma [71].

## 10. Conclusions

With the burgeoning epidemics of asthma and obesity in the United States and the scientific evidence that these two conditions are intimately related, clinicians caring for individuals with these disorders will see a dramatic coincidence of the two, in either of the two identified asthma-obesity phenotypes. Distinguished by age-of-asthma-onset, which typically correlates with atopy, the two described asthma-obesity phenotypes (early- versus late-onset asthma) have varying clinical presentations that can aide clinicians in directing their management. Early-onset obese asthmatics have a more severe clinical presentation, which is also characterized by atopy and eosinophilic inflammation. Late-onset obese asthmatics do have more severe asthma than their lean counterparts, but are distinguished from the early-onset obese group by having a lower incidence of atopy and eosinophilic inflammation, and this phenotype is more prevalent among females. In both asthma-obesity phenotypes, however, excess weight negatively affects asthma control, severity, and medication response. Armed with this knowledge, we must provide a more holistic approach at this point in time, offering weight loss reduction strategies in addition to standard asthma care to individuals who fall into both asthma-obesity phenotypes. 

Future research in this area should distinguish the subject populations in terms of age-of-asthma onset to provide a more rigorous assessment of the inflammatory fingerprint, medication response, effects of weight loss, and new therapeutic strategies in these apparently differing asthma phenotypes. Since the majority of standard asthma controllers have been found to be less effective in the obese state, there must be a drive to evaluate other possible therapies, perhaps focused on a more metabolic target, in order to adequately control these more severe phenotypes of asthma. Furthermore, obesity itself is a heterogeneous disorder and its variations may alter its effects on asthma. Future studies may be able to dissect how different forms of obesity (based on severity, android versus gynoid, childhood-onset versus adult-onset) may affect asthma phenotypes. Lastly, nutritional studies, which have been reviewed by Lang [72], rather than assessments of only obesity, may offer further insight into how an individual's intake affects varying asthma phenotypes.
